# Perceived benefits and barriers to the use of long-acting injectable antiretroviral treatment among adolescents and young people living with HIV in Western Kenya: qualitative findings from the KuwaFree! LiveFree! Study

**DOI:** 10.3389/fmed.2025.1518719

**Published:** 2025-03-07

**Authors:** Shukri A. Hassan, Dennis Munyoro, Mehar Maju, Whitney Biegon, Salim Bakari, Eunice Kaguiri, Anjellah Jumah, Mark Omollo, Valerie Obare, Caitlin Bernard, Edith Apondi, Edwin Were, Rena C. Patel

**Affiliations:** ^1^Department of Medicine, University of Washington, Seattle, WA, United States; ^2^Academic Model Providing Access to Healthcare (AMPATH), Eldoret, Kenya; ^3^Ministry of Health, National AIDS and STI Control Program (NASCOP), Nairobi, Kenya; ^4^Department of Obstetrics and Gynecology, Indiana University School of Medicine, Indianapolis, IN, United States; ^5^Department of Reproductive Health, Moi University College of Health Sciences, Eldoret, Kenya; ^6^Department of Medicine, University of Alabama at Birmingham, Birmingham, AL, United States

**Keywords:** HIV, long-acting injectable antiretroviral, adolescents, antiretroviral therapy, low-and middle-income countries, Kenya

## Abstract

**Introduction:**

Adolescents and young people living with HIV (AYPLHIV) face significant hurdles in adhering to daily oral antiretroviral therapy (ART). Long-acting (LA) ART, such as injectable cabotegravir and rilpivirine, may help overcome these hurdles. However, little is known about the perceived benefits and barriers to LA ART usage by AYPLHIV in resource-limited settings.

**Methods:**

We conducted focus group discussions (FGDs) with four target groups of adolescents/youth, providers, policymakers, and other advocates in western Kenya from November 2021 to April 2022. The FGDs elicited participants' thoughts on LA ART implementation in Kenya, particularly the benefits and barriers of LA ART use amongst AYPLHIV. Our analysis combined both inductive and deductive approaches, beginning with open coding of the data, then organizing them in predetermined socio-ecological model (SEM) domains.

**Results and discussion:**

We conducted a total of seven FGDs with 58 participants across four stakeholder groups: AYPLHIV (2 FGDs, *n* = 14), healthcare providers (2 FGDs, *n* = 19), health/youth advocates (2 FGDs, *n* = 16), and policymakers (1 FGD, *n* = 9). We identified several benefits, largely centered around the individual and interpersonal level, as well as barriers, largely centered around the health systems levels. Participants viewed LA ART as a welcome alternative to oral ART due to benefits like improved adherence, reduced pill burden, increased convenience, enhanced privacy, decreased stigma, lower risk of accidental disclosure, and convergence in using LA contraception. At the interpersonal level, LA ART was valued for furthering relationships, especially for AYPLHIV (e.g., dating). At the health systems level, LA ART expanded first-line treatment options. Conversely, barriers to health systems integration included conflicts with service models, increased clinic burden, supply chain issues, and waste disposal. Individual-level barriers included fears of injections, side effects, concerns about a new drug, and reduced effectiveness if mixing LA ART with oral ART.

**Conclusions:**

The perceived benefits of LA ART for AYPLHIV, centering on individual and interpersonal levels of positive impacts, appear in tension with the anticipated barriers for health systems readiness in Kenya. While the prospect of offering LA ART is overwhelming positive, country programs will have to invest in health systems readiness before rolling out LA ART.

## Introduction

Kenya, a priority President's Emergency Plan for AIDS Relief (PEPFAR) country, faces significant challenges in supporting adherence to HIV treatment for adolescents and young people living with HIV (AYPLHIV), aged 15–24 years, a sizable group worldwide on HIV treatment (1). In low-and middle-income countries (LMICs), AYPLHIV, especially adolescent girls and young women living with HIV (AGYWLHIV), must navigate not only the burden of avoiding HIV transmission but also complexities such as preventing unintended pregnancies, protecting against sexually transmitted infections, and, overall, accessing comprehensive sexual and reproductive health services (2, 3). AYPLHIV, particularly in LMICs, lag behind the 3^rd^ 95-95-95 UNAIDS goal of viral suppression (4, 5). In Kenya, AYPLHIV have lower rates of ART adherence compared to other age groups, facing unique challenges in adhering to current daily oral ART (6). Specifically, AGYW also face unique needs for co-delivery with family planning to prevent unintended pregnancies, including significant barriers in adopting and continuing long-acting contraceptives, facing gender-based violence, and poor mental health (2, 3, 7, 8).

The introduction of the first complete long-acting (LA) formulation of an antiretroviral therapy (ART), injectable cabotegravir and rilpivirine, has generated significant enthusiasm globally. Approved by the U.S. Food and Drug Administration in March 2022, this LA ART is anticipated to substantially impact HIV treatment practices by enhancing patient adherence, persistence, and providing an alternative for individuals who struggle with oral ART (9, 10). LA ART offers treatment options for patients and providers, which is especially beneficial in high-burden contexts like Kenya.

LA ART could, for example, enhance ART adherence by improving privacy, reducing social stigma, and increasing convenience, independence, and emotional wellbeing through eliminating the need for daily pills and frequent clinic visits (11). A recent study indicated a high willingness among participants to use injectables for HIV treatment due to the convenience provided (12). Additionally, the CARES study in sub-Saharan Africa demonstrated the feasibility of coupling LA ART with the public health approach to treatment monitoring in LMICs, i.e., checking for viral suppression every 6 months (13). Additionally, evidence from studies, like the LATITUDE study, demonstrates the effectiveness of LA ART in individuals struggling with viral suppression in high-income countries (HICs), suggesting potential benefits for AGYW who face adherence challenges (14, 15). Recognizing these benefits, studies have specifically recruited AGYWLHIV to assess the appropriateness and effectiveness of LA ART (16, 17).

Despite the potential benefits of LA ART, barriers exist for its rollout in both HICs and LMICs. Existing hurdles in HICs, such as cost, insurance pre-authorization processes, workflow integration, and provider training, can be exacerbated in LMICs due to resource constraints (18–22). Beyond these health systems readiness barriers, LA ART adoption in LMICs may be hindered by anxieties about safety, effectiveness, and the increased frequency of clinic visits compared to oral ART regimens (21, 22). In LMICs, these issues are compounded by widespread distrust of the healthcare system, particularly among communities disproportionately affected by HIV.

This study aimed to investigate how these various barriers and facilitators might manifest in the specific context of Kenya, a high HIV burden, PEPFAR-priority country with a robust public sector ART program. By understanding these contextual factors, we can develop strategies to optimize the rollout of LA ART in LMICs, and maximize its potential benefits while minimizing barriers to its rollout. Our study makes a distinct contribution to the literature by offering a comprehensive, multi-stakeholder perspective on the implementation of LA ART among AYPLHIV in Kenya. Unlike prior research, such as Toska et al. in South Africa and Zakumumpa in Uganda (23, 24), which primarily examined barriers from the perspectives of either patients or healthcare providers, our study integrates insights from AYPLHIV, healthcare workers, youth advocates, and policymakers. This broad approach allows us to capture not only individual and health system barriers but also the interpersonal and social dimensions influencing LA ART uptake. Additionally, our study uniquely explores the gendered dynamics of LA ART, particularly how it impacts privacy, stigma, and treatment adherence among adolescents and young people of all genders in Kenya. Furthermore, while previous studies primarily focused on clinical and logistical challenges, we emphasize the specific health system integration barriers in Kenya, such as service delivery conflicts, clinic burden, and supply chain constraints, which are critical to informing policy and programmatic strategies for LA ART rollout. By incorporating these diverse perspectives and a country-specific lens, our study provides novel insights that advance the understanding of LA ART implementation in sub-Saharan Africa.

## Methods

### Study setting, study team, and positionality

This study was conducted in Eldoret, Uasin Gishu County in western Kenya, where the last estimated HIV prevalence was 3.74% in 2023 (25). In the region, while adolescent AIDS-related mortality saw a 49% reduction between 2015 and 2022, new HIV infections among those aged 15–24 climbed 40% during the same period (26).

This study was led by Moi Teaching and Referral Hospital (MTRH), the study site, and University of Washington academics. MTRH is home to the only youth-friendly, integrated HIV treatment and family planning clinic, named Rafiki Clinic, in Uasin Gishu county. Established in 2016, Rafiki Clinic provides comprehensive services, including HIV care, family planning, sexually transmitted infections screening, and mental health services. It also conducts outreach targeting street-connected youth, orphans, and vulnerable children, while also serving as a hub for extracurricular and social activities.

The study team comprised academic researchers, study staff, and youth peer educators. The majority of the 13 team members were Black Kenyans, except for four individuals: two South Asian Americans, one White American, and one Somali American. It is important to note that none of the individuals residing outside of Kenya were of Kenyan origin (though some had extended family members living in Kenya). One of the three peer educators was female. Throughout all phases of the project, including planning, execution, analysis, and dissemination, the team regularly met and generally utilized a consensus-driven approach for decision-making. Non-Kenyan team members collaborated closely with Kenyan counterparts to ensure that the results and interpretations aligned with the perspectives, beliefs, or lived experiences of the Kenyan team members.

Our study reporting follows the Consolidated Criteria for Reporting Qualitative Research (COREQ) (27), and the checklist is available in the Supplementary material. This study underwent ethical review with the Institutional Research and Ethics Committee at Moi University in Kenya (0003912) and Human Subjects Division at the University of Washington in the United States (00012640).

### Data collection

#### Overall approach

Our qualitative data collecting strategy consisted of focus group discussions (FGDs) with four key groups in western Kenya: (1) AYPLHIV, both females and males, (2) health care providers, (3) policymakers, and (4) advocates. The FGDs were performed between November 2021 and April 2022. While we aimed to conduct two FGDs per group, we conducted only one with policymakers due to scheduling conflicts and limited availability of participants at both the national and county levels. The FGDs elicited participants' perspectives on the integration of injectable LA ART in Kenya, focusing on the perceived benefits and barriers of LA ART usage among AGYWLHIV.

#### Sampling

We recruited FGD participants using purposive and snowball sampling, guided by the study team, particularly youth peer educators, on recruitment and target participants. We aimed to recruit participants across the four categories of relevant stakeholders named above. To recruit the AYPLHIV and providers, we used flyers and word-of-mouth to identify an initial round of potential participants. Then, we asked those potential participants to recommend 1–2 additional potential participants. The AYPLHIV were identified and recruited from the Rafiki Clinic during their scheduled appointments by research staff. Providers were recruited through the facility leadership, identifying potential participants from Rafiki Clinic and other HIV and family planning care clinics at the study site, all of whom were directly involved in HIV care. To recruit advocates and policymakers, we identified key groups and cadres for inclusion and contacted them to provide representatives from their organizations who were willing to participate, and to recommend 1–2 other groups or team members for us to invite.

#### FGD procedures

Before conducting the FGDs, we obtained oral informed consent in English or Kiswahili, per the participant's preference and administered a brief survey on paper (later entered in REDCap by study staff).

The survey covered demographic data including age, gender, education, and experience with HIV treatment for AYPLHIV, or their role, area of service, and length of experience for policymakers, advocates, and healthcare providers (see Supplementary material for survey). Each participant received a monetary payment of Ksh1100 (~US$10) for time and travel. Peer mentors were involved in organizing the FGDs to ensure that the sessions were lively and youth-friendly.

FGD facilitators already working at the study site were chosen based on experience conducting FGDs and working with AYPLHIV. Facilitators conducted the FGDs in the participants' chosen language, while another Kenyan member of the research team took short discussion notes. The FGDs were conducted both in-person and virtually using the Zoom platform. The in-person FGDs, which included sessions with AYPLHIV and healthcare providers separately, took place at Rafiki Clinic. In contrast, the FGDs with advocates and policymakers were held virtually via Zoom. The recorded audio from all sessions was then translated and/or transcribed into English. The short notes were typed directly into English. We used the transcription and translation services within the qualitative research core at study facility, and the final transcripts were reviewed for accuracy by the respective FGD facilitator. The transcripts were not sent to any of the FGD participants for checking accuracy.

#### FGD guides

We developed the initial FGD guides using existing literature and team knowledge. The FGD guide largely focused on deepening understandings of perspectives, feelings toward, and choices of people living with HIV in regards to LA ART and family planning. The guides covered the following four main domains: (1) living with HIV as an adolescent or providing care, policy-making, or advocating for such individuals; (2) perspectives on adolescents using family planning; (3) perceived benefits and barriers of LA ART; and (4) health systems readiness for large scale LA ART rollout. We used the socio-ecological model (SEM) *a priori* to help us organize subthemes and probes within each domain (28, 29). Additionally, for the domain pertaining to large scale LA ART rollout, we used the PRISM (Practical, Robust Implementation and Sustainability Model) framework (30). Each FGD for the four target groups was modified for content to be most pertinent to that group (e.g., for AYPLHIV we asked their first-person experiences, while for providers, we asked about their observed experiences of AYPLHIV). Reflecting on the conduct of each FGD and insights gained from the FGD short notes, we iteratively improved on the development of subsequent FGD guides for both that group and other groups. The guides can be found in Supplementary material.

### Data analysis

**Figure 1:**
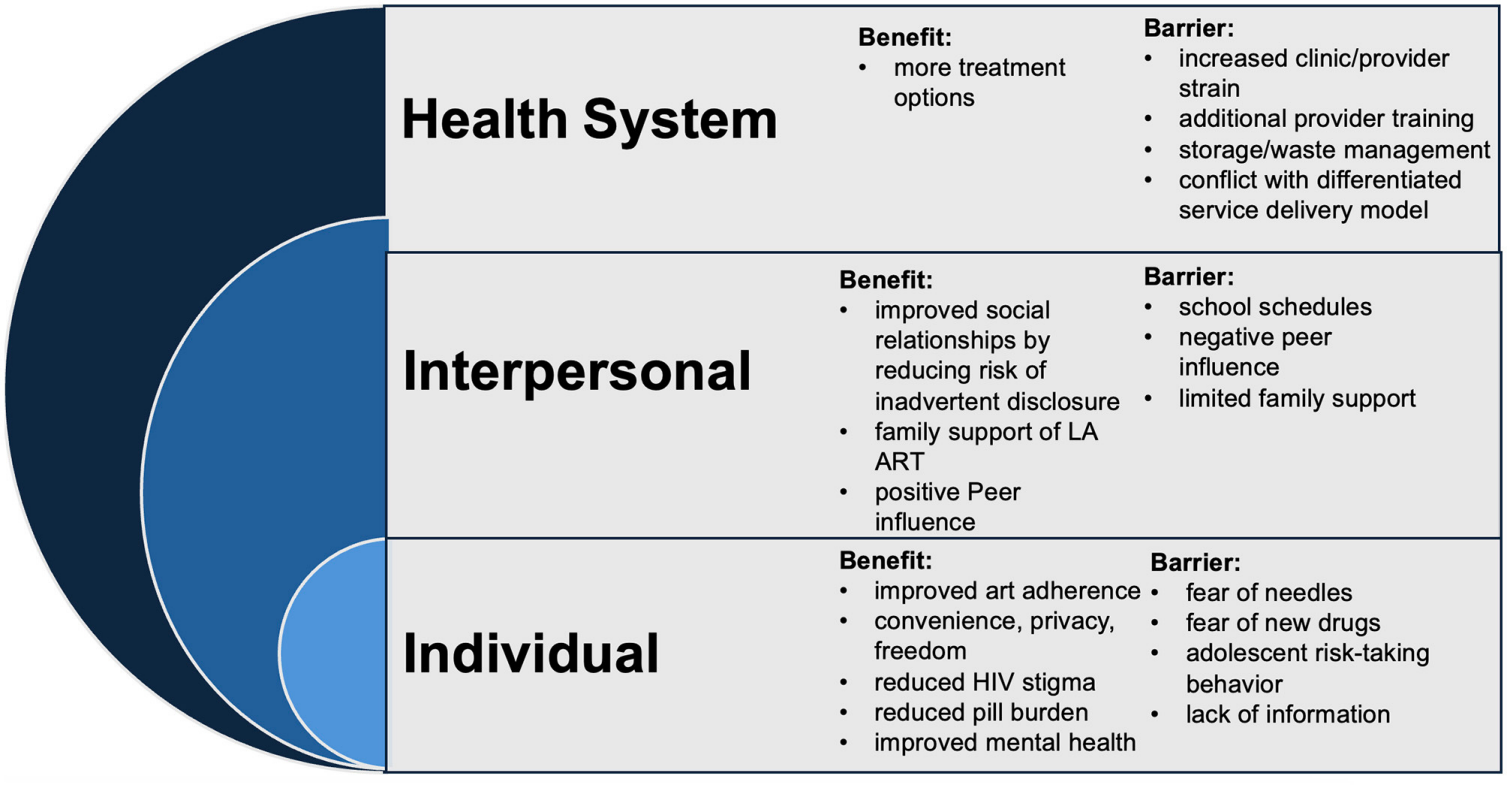
Socioecological model of perceived benefits and barriers to long-acting injectable antiretroviral treatment (LA ART), KuwaFree! LiveFree! study, Kenya, November 2021–April 2022 (7 focus groups discussions, *n* = 58 participants).

We utilized the qualitative analysis software Dedoose (Version *9.0.17*, 2021) for coding 2/6/25 1:19:00 PM. We utilized both inductive and deductive approaches overall to data analysis, with largely inductive coding and deductive thematic analysis; this dual approach enabled us to capture participant-driven insights while situating our findings within broader contextual and systemic levels, providing a comprehensive understanding of the experiences and barriers associated with LA ART among AYPLHIV. We largely used inductive coding, allowing the research team to identify emergent themes directly from the data without being constrained by predefined categories. Four research assistants (DM, SB, WB, and MM) performed the coding under the direction of one study team member (SAH) and primary investigators (RP, EW, EA, and CB) through weekly meetings and iterative feedback from this broader research team. SAH prepared an initial codebook based on the FGD guides and her assessment of the first few transcripts. Under the guidance of SAH, the four research assistants coded the remaining transcripts and iteratively adjusted the codebook as transcript coding continued. In the first round of coding, all four coders collectively coded one transcript in a group setting at the same time, with any discrepancies in coding resolved by consensus. In the next round of coding, two coders separately coded a single transcript, and then the group met to resolve any discrepancies. In the third round, the remainder of the transcripts were divided for primary coding among the four coders, and each coded the remaining assigned transcripts alone, with another team member (SAH) reviewing and double-coding all transcripts. After coding, the team held a 2-day long, intensive in-person analysis session in Kenya. SAH and three Kenyan research assistants used thematic analysis (31, 32) to keep an analytic codebook that grouped our codes into overarching domains with subsequent themes, which were later grouped by the SEM by categorizing themes according to the four levels of the model (individual, interpersonal, organizational/institutional, and policy) (Figure 1), with convergent and divergent subthemes and illustrative quotes. We used this codebook to develop analysis memos leading into separate manuscript efforts. Of note, we conducted participant “member check” or vetting of our analysis and findings via dissemination meetings held in-person from July through November 2022 with each of the four FGD groups. All participants were explicitly invited to join these meetings where study staff presented the findings via a prepared presentation, and we elicited participant feedback. One study staff took notes during these sessions, which were also included in our analysis to help underscore emphasis or highlights.

## Results

We conducted seven focus group discussions with a total of 58 participants across four key stakeholder groups: (1) AYPLHIV, (2) healthcare providers (including peer mentors), (3) health or youth advocates, and (4) policymakers, with all groups except the last having two FGDs per group (Table 1).

**Table 1:** Participant characteristics for the focus group discussions in the KuwaFree! LiveFree! study, Kenya, November 2021–April 2022 (7 focus groups discussions, *n* = 58 participants).

**Variable**	**Category**	**N (%)**			
**Gender**		**Adolescent and youth (*****n** =* **14)**	**Healthcare providers (*****n** =* **19)**	**Health advocates (*****n** =* **16)**	**Policy makers (*****n** =* **9)**
	Female	7(50)	12(63.2)	10(62.5)	6(67.0)
	Male	7(50)	7(36.8)	6(37.5)	3(33.0)
Age in year		Below 18 −7(50)	25 and below −5(26.4)	Below 25 −7(43.9)	Below 40 −3(33.3)
		18–21 −6(42.9)	26–35 −9(47.5)	25–30 −6(37.7)	40 and above −2(22.2)
		Above 21 −1(7.1)	Above 35 −5(26.5)	Above 30 −3(18.9)	Missing 4(44.5)
Highest level of education	Secondary	11(78.6)	-	-	-
	Some college or higher education	3(21.4) 0	-	-	-
Marital status	Single	14(100)	-	-	-
Occupation		Not employed 11(78.6%)	Clinical Officer −6(31.6)	-	-
		Peer educator 1(7.1)	Nurse −1(5.3)	-	-
		Student 2(14.3)	Nutritionist −1(5.3)	-	-
			Peer mentor- 8(42.1)	-	-
			Research assistant-2(10.5)	-	-
			Social worker-1(5.3)	-	-
Services accessed/offered	HIV treatment	Type of services accessed at the clinic	Type of services provided at the clinic	Thematic focus of advocacy activities	Area of service
		14(100)	14(73.7)	3(18.8)	2(22.2)
	HIV prevention	0	13(68.4)	11(68.8)	2(22.2)
	FP services	0	10(52.6)	5(31.3)	0
	Adolescent and youth	-	-	12(74.1)	1(11.1)
	Other services	0	12(63.2)^*^	5(31.4)^**^	2(22.2)
Duration Since ART initiation (years)	≤ 10	2 (14.2)	–	–	–
	11–20	6(42.6)	–	–	–
	21+	1(7.1)	-	-	-
	Unknown	4(28.6	-	-	-
Length of time worked			Length of time in the position	Duration worked in the organization	
		-	< 1 year −3(15.9)	< 2 years −1(12.5)	-
		-	1–5 years −6(31.7)	2–3 years −3(37.5)	-
		-	>5 years −10(52.9)	>3 years −4(50)	-
Role in organization	Program implementer	-	-	5 (18.8)	3(33.3)
	Service provider	-	-	4(25)	
	CBO	-	-	9(56.3)	
Sector engaged	NGO	-	-	10(62.5)	
	None	-	-	2(12.5)	
	Private	-	-	3(18.8)	
	Public	-	-	1(6.3)	5(55.5)

FP, family planning; ART, antiretroviral therapy; CBO, community-based organization; NGO, non-governmental organization. ^*^Adherence counseling, monitoring viral load, helping in financial support, pediatrics research, retention, training on stigma and disclosure and HIV care, treatment of comorbidities, nutrition education and counseling. ^**^Key populations, mental health promotion.

For the AYPLHIV group (*n* = 14), half were female and under 18 years old, had been on ART for 11+ years, and were mostly in secondary school; none were married. Healthcare providers (*n* = 19, 12 female) included peer mentors, doctors, nurses, nutritionists, and social workers. Most had over 5 years of experience in an HIV clinic and involvement in ART or family planning provision. Advocates (*n* = 16, 10 female) represented various advocacy areas, including HIV prevention, family planning, youth services, key populations, and mental health. Policymakers (*n* = 9, 6 female) primarily worked within the HIV public sector. Despite variations in age and professional backgrounds across these groups, responses in the FGDs were generally similar. However, when dissimilar, we emphasize any distinctions that arose.

Below, we detail the two overarching themes of perceived (1) benefits and (2) barriers of LA ART, organized by the socioecological model levels (i.e., starting with the individual level going up to policy/cultural), with detailed nuances captured under each subtheme. Occasional example quotes are included in-text and additional quotes can be found in Table 2. We note that while the current formulation of LA ART, injectable cabotegravir/rilpivirine, is available for every 1- or 2-month injections, our results text refers to it as a “monthly” option for simplicity. We also note specific nuances when more relevant to AGYWLHIV.

**Table 2:** Main themes, subthemes, and example quotes for perceived benefits and barriers to LA ART use, KuwaFree! LiveFree! study, Kenya, November 2021–April 2022 (7 focus groups discussions, *n* = 58 participants).

**Main theme**	**Subtheme**		**Quotes**
**Benefits to LA ART integration**	Individual level	*Improved ART adherence*	“*So to me I think it is a good one because first of all who would want to take the pills every day? It is going to help in adherence.”* **Healthcare Provider FGD 1** “*Yes. And things to do with disclosure is going to being left behind. I am not so sure that is a good thing because one thing I am sure about is it is going to have a good adherence especially in our adolescents we have them going to school and so if we are going to have the monthly injection, to me that is a good thing, it is going to improve on adherence, it is going to reduce pill burden and to me adherence is going to go quite well.”* **Healthcare Provider FGD 1** “*I think the percentages in terms of the adherence, there will be an increase. Secondly I think the issue to do with stigma in schools for those who take the pills will be a thing of the past”* **Healthcare Provider FGD 1** “*You like going to parties but them your medication time is really not considerate for you. So you find this adolescent will default from getting the medication because they want to go for that. The party starts at 6, you are taking medication at 10 o'clock. So I have to leave my medication in the house to attend this party because that is the priority to them-I am not feeling sick, so my priority is my friends and I cannot tell them that I cannot come to a party because I have medication to take. So I am feeling it is going to get a lot of positive responses and acceptance.”* **Healthcare Provider FGD 1** “*It will also help on missing of doses. You know there are those who forget to take the pills. You don't have to take it regularly and so you don't have to keep time.”* **Adolescent Youth FGD 3** “*Actually for me the injection is far much better because you know carrying drugs when going to school becomes tricky especially in terms of storage, going with oral pills to school. You find a lot of adolescents when they reach boarding schools, they tend to stop taking the drugs at home because they are going to school and they don't want to disclose to their friends. So you leave the drugs during that period when you are in school and then you take the drugs during holidays. You get me. But now with injections because you come and get injected, you don't have to carry any medication. So it is easier to manage in schools and you don't have to disclose every time unless it is someone who is giving you permission or something.”* **Adolescent Youth FGD 3** “*It is good because you will not keep on taking the drugs. You will be 100% sure that you are in LDL. Like you see sometimes if you have been taking your drugs at 7 and then maybe you had gone somewhere and you come back late, and when you go and take your LDL, you will not be sure you are on LDL but with injection you are sure you are on LDL.”* **Adolescent Youth FGD 4**
		*Reduced HIV Stigma, Convenience, Privacy*	“*For me, it is somehow going to help in terms of privacy. Like on a daily basis you have to take your pills, maybe with your friends it is sometimes very hard but if you are going to get those injectable, at least it will boost the privacy because one cannot know it easily. You will be getting injected monthly and so you just go once. You get injected once and then you wait for another month and all that.”* **Adolescent Youth FGD 3** “*Yeah, every day. Maybe you can have your friend or your cousin. So they will come and stay with you for a duration of time and so you cannot be hiding today, tomorrow, the day after. Even if you hide yourself today, tomorrow or the day after or the other time they will try asking you; ‘where do you go to when it reaches this time?” at least with the injection it will try to boost your privacy.”* **Adolescent Youth FGD3** “*The ones in boarding will prefer injection obviously for freedom. You can do anything, for preventing stigma. You know if the other friends find out, obviously there will be stigma.”* **Adolescent Youth FGD 3** “*For boarding, I will prefer injection because during the time that when you want to take the drugs, there are those people who investigate you up and down what you are taking. But for the injection, no one will know. You are just there, you are free.”* **Adolescent Youth FGD 3** “*And then again you can also travel like she has said. You can go for sleep overs [Laughing…] like you see it is difficult for me to leave my home and go somewhere because of the medicine. I do not have that privacy in other places. But if I get injected like once in a month…”* **Adolescent Youth FGD 3** “*But for adolescents it is all about…you know the stigma is still there. So, if people don't see me taking treatment, then they are not going to judge me I look like I have HIV because sometimes when people know you have HIV, they start looking at you like; ‘you have never been looking like you have been having HIV'. So, there is also that. I think the stigma as well as the pill burden as well as the issues of discretion that comes with the injectable, I think that is what makes it attractive to young people.”* **Adolescent Youth FGD 3**
		*Reduced pill burden*	“*You know going to parties or going to travel, so carrying medications sometimes loses its potency because walking around with the bottle, we call it ‘kayamba' when you put it in the bag it is like ‘chaka- chaka- chaka- chaka' so it will be easier for those people who travel a lot*.” **Healthcare Provider FGD 1** “*I feel it is going to get a very good response because a lot of adolescents really get tired with taking medication especially most of the young people coming to Rafiki are people who were on medication for a longer time, let us say from during birth, from 2 years to right now. So most of them, in my experience talking with the adolescents most of them feel like the reason the adolescents take drug breaks is because taking medication daily, a person becomes tired. Even malaria tablets most people don't finish it, right?”* **Healthcare Provider FGD 1** “*So apart from the reduction in the pill number, you know just getting a jab and you go home, nobody really knows what you are going to the hospital to do. It is better as compared to taking the oral pills”* **Healthcare Provider FGD 1** “*Others tell you ‘I forgot my meds when I was traveling somewhere' or I got an abrupt assignment and I just had to leave without drugs. So those ones will really be advantaged because the drug will still be in the system and they don't have to worry about adherences.”* **Healthcare Provider FGD 2** “*I also think that the school going would really prefer this method rather than those who are maybe at home because you realize that when they are in school, they really feel like taking every day pill and maybe their school mates are seeing them, the matron is seeing them looking for the dorms' key might be a challenge. I think they would also prefer the injectable because it does not involve them taking the pills every day.”* **Healthcare Provider FGD 2** “*Personally for me I prefer the injection because it reduces the pill burden. You know you could be hustling or you are in town, as a youth you also need to fend for yourself. So during that time when you are doing your activities, sometimes you tend to forget your medication. You may not get time and such things.”* **Adolescent Youth FGD 3** “*According to me I don't see any preference on oral pills because it is tiring taking drugs. Not everyone finishes even the malaria pills and it is a three or four day thing. So taking those drugs daily is tiring. But now if you imagine taking drugs daily at a specific time or getting one injection once a month. I will definitely go with once a month and forget it until the next month.”* **Adolescent Youth FGD 3**
		*Improved mental health*	“*In my opinion, I have heard like number 1 and number 2 have said, I have heard it reduces stress. You see most people are stressed, when you go taking your drugs, you are thinking about your status. So I have heard that the drug can reduce that because one will not be having that mentality so much.”* **Adolescent Youth FGD 4** “*Yeah, so I see the rate of depression will reduce. So if you look at the statistics, it will reduce that mentality. It will reduce the mental health and depression. You will just be thinking that when it is end of the month, you get that injection, it is like salary and you will be going to get that salary. You get that injection and you will not have that mentality. Your brain will be used to do many other things including your job work, your school work.”* **Adolescent Youth FGD 4**
		*Potential for integration with family planning services*	“*For me I can say it will be of benefit because it is two services at the same time. So you find you are really easing work for this client.”* **Healthcare Provider FGD 2** “*But integrating family planning into an HIV clinic makes sense because, once you're diagnosed with HIV, you don't have a choice about treatment—you must take your medication. However, with family planning, you have a choice whether to use it or not. The number of people attending the HIV clinic might not be as large as those coming for family planning. So, integrating family planning into the HIV clinic could help.”* **Policy Maker FGD 6** “*Because most of the youth are found in HIV clinic other than the other one. So you will find that most of the youths who are found in the clinic are the same, same ones going to take the family planning.”* **Adolescent Youth FGD 3** “*One thing is clear: we still have a major issue with the uptake of contraceptives, especially long-acting ones. Many of the people using long-acting contraceptives tend to be older women, typically those who have already given birth and don't want more children for a while. So, I think if we're offering injectables for ART (antiretroviral therapy), we should also bring the conversation about contraceptives into the mix. This way, young girls can make informed decisions about what works best for them.”* **Advocate FGD 5** “*I would say that an adolescent already using long-acting family planning could be influenced to opt for the long-acting injectable for ART. If they've been benefiting from just one shot and then not having to worry about keeping track of a schedule, that convenience could encourage them to choose the long-acting injectable for ART as well.”* **Advocate FGD 5**
	Interpersonal level	*Improved relationships by reducing risk of inadvertent disclosure*	“*I see longer acting ART as a very good thing although there are challenges as has been said. It will avoid accidental disclosure. For example if I am coming for my jab here in Rafiki and coincidentally I find my boyfriend here, I think the doctor will cope up with me and say this is a malaria injection. So at least. Also for those people who avoid disclosure, it is the best part of it. Is there any need to disclose if I am taking a monthly injection? No. there is no evidence that shows I am using ARVs. Only the doctor and I are supposed to know.”* **Healthcare Provider FGD 1** “*Those in colleges, it is themselves. If they want to go out, it will be easier to go out with friends and do whatever.”* **Adolescent Youth FGD 3** “*The one in a relationship should prefer an injection one because the moment you want to go and see the other person there, you will not have to carry the packages. So at least you have been injected and yet your boyfriend doesn't know your current status. So the injection is okay.”* **Adolescent Youth FGD 3** “*Because someone may appear, a friend may appear and tell you there is a bash today, let us go and then you are there going. When you get there, maybe the bash gets exciting and then there is no way you will leave the people saying that you are going to take your medication.”* **Adolescent Youth FGD 3** “*For me I feel like there might be a disparity when it comes to how it affects all adolescents because adolescents who are in relationships and have not disclosed will love the injectable.”* **Health Advocate FGD 5** “*the advantage is, you find for example adolescents who are out of school or in campuses and are dating, the problem of keeping your medication where no one can find. You will have eliminated that one. So accidental disclosure amount other adolescents. That will be one thing.”* **Healthcare Provider FGD 1** “*Just to add on what number 1 has said, basically it will help unnecessary disclosure because with disclosure, nowadays there is Google. Someone can Google your drugs and then gets to find out that this is ART. But with injections, when you come to the clinic you only get injected once and you don't have to carry medications. So that will help with unnecessary disclosure that you have not planned for.”* **Adolescent Youth FGD 3**
		*Family support of LA ART*	“*I think what will influence those who are in primary school to take the either the injection is the guidance from the parent because obviously they are not independent. So the parent will influence. I think also for those who are in secondary*.” **Adolescent Youth FGD 3**
		*Positive peer influence to switching to LA ART*	“*At that point of rolling out you may not find a lot of adolescents because most of them are as number 5 has said-the fear of uncertainty, who has been tested, how did it react with people. But then going forward, you will find a lot of people who might even overwhelm even the study because let us say if you were targeting 100 people, as soon as you have given the first 20 or 30 and they are doing okay, you will find that the other 80 will exceed to even 500 wanting the injection because the 20 have taken the injection and nothing has happened. So you will have to put that on a beam balance to see how you are going to balance. You will find that people will fear it at first, just as the way second line was. A lot of people didn't want to go on second line because they feared. You would hear stories that you will diarrhea for 6 months and such things and people feared it. But as soon as it was explained and some few adolescents got into second line, other so that it is not that bad as we thought.”* **Adolescent Youth FGD 3** “*The first times of the study you will find it does not have much demand but as soon as a few adolescents have taken it and they are doing well…adolescents do stories ‘I am on injection. I get injected once a month' ‘how is it reacting for you? Do you have headache?' ‘Do you have this?' ‘No, no' ‘let us go.' So you will find that a lot of adolescents will come later with a lot of them wanting to join the study and it may overwhelm.”* **Adolescent Youth FGD 3** “*It will be like a motivation thing to the youth because if you give someone with a low viral load, this one will ask; ‘why am I not being given?' the reason behind it is that he/she is not virally suppressed. So if you tell him/her that if you improve your viral load, you will be able to acquire the injection for example if it is once a month or depending on how the injection will be and also that injection is not a permanent treatment for HIV.”* **Adolescent Youth FGD 3**
	Health Systems/Institutional level	*Increase in clinicians first line options*	“*Case in point, we had an opportunity to have training with the university abroad and we just learnt that the choices of first line for their clients is not really dependent on the guidelines that they have but the choice lies with the clients because they have a variety to choose from. Unlike a country which is categorized as middle income country where we have limited choices. So if we reach such a place where the variety is wider, then I think we will be able to serve our clients better.”* **Healthcare Provider FGD 2**
**Barriers to LA ART Integration**	Individual level	*Fear of needles*	“*I am feeling like 100% acceptability unless those people who fear injections. They fear it, especially people living with HIV. We have been growing in injections almost every now and then. Injections for viral load, whenever you are sick you want to go for”* **Healthcare Provider FGD 1** “*We might experience under dose among people because we will give people the lab order but they will not go to the lab. You find there are people who are afraid of the needle and they are supposed to take that needle plus another drug. They will not take the injection, they will just take the drug only. So you will find there is under dose to some people who don't prefer that injection.”* **Healthcare Provider FGD 2** “*It is painful.”* **Adolescent Youth FGD 3** “* Such circumstances come in especially for the children and those people who fear the injection. There are those people who do not go one way with injections. They prefer orals. That is where point number one comes through so that it becomes a choice to those who don't prefer injections, they can just use the pills. And then definitely you know the young children don't get along with injections. So I am feeling it will still be a challenge to them.”* **Adolescent Youth FGD 3** “*At a personal level, at first I will be expected to get an injection that it becomes a lifestyle having issues that I have to take an injection at a certain time. It might turn out to be a bit traumatizing.”* **Health Advocate FGD 5**
		*Fear of side effects*	“*And maybe to add on what she has said, maybe the fear of the side effects that might come with the injection so somebody would reason that if I take a drug that has severe side effects, I am able to stop that medication. But now with the injection, once it is injected, it is already in the system and so I have to bear with that”* **Healthcare Provider FGD 2** “*For me I might feel like maybe people are different. Maybe someone will get injected and it reacts with their body, others may have skin rashes.”* **Adolescent Youth FGD 3** “*Yes, the fear of accepting it. You don't know, has it ever been tested on anyone else? That is the first thing I will ask for me. This is because maybe my body will react very differently. Maybe I will become sick. So I will need to know first if it has been tested on anyone.”* **Adolescent Youth FGD 3**
		*Fear of new drugs and LA ART reduced efficacy*	“*Also the fear of a new thing being rolled out just as the covid vaccine came. There was that fear of how this is going to be. But now you see there is an improved uptake of the vaccine. So maybe that fear.”* **Healthcare Provider FGD 2** “*Maybe the injected dose was not enough and may get finished before the month ends. The viral load will go up and definitely I will become sick.”* **Adolescent Youth FGD 3**
		*Dislike for increased clinic visits*	“*I also agree because when the patients ask about the injectable, they thought it will take around 3–6 months then they come for another injection. So when I told them it is monthly, they said ‘I would rather continue with my pill”'* **Healthcare Provider FGD 2**
		*Exploration and Risk-Taking behaviors*	“*For us adolescents we really like to explore. The way number 4 has said, I will be using a jab and I will be thinking; what if I use…if I have missed a jab for two days and so let me take two days' tablets and then when I go to clinic I will say I didn't take any medication. So that is the kind of challenges we will get.”* **Adolescent Youth FGD 3**
		*Lack of accurate LA ART information*	“*You know currently most people are reluctant, they don't know anything about this medication. We have just heard there is injection but we don't know anything about it. but now if you give information and education that this medication is there, it has reacted like this, it has been tested here, these are the reactions, possible side effects and such things, you will find that it will become easier to take it because all the question he/she had no answers, he/she will have all the information about the medication. So if you give that information that this is how this drugs reacts, for instance February has 29 days. So how do you quantify a month? Is it 30 days, or 28 days? If you address such things, allot of people will get to understand this is how this medication has worked and this is the possible side effect, the same as covid vaccine is doing. So you give people information first. Definitely there will be myths out there. But as soon as people get the right information about the vaccine, they will come and take it.”* **Adolescent Youth FGD 3** “*Maybe when the adolescent visits every time you inform about the monthly injection so that they get to learn and know more. Like in Rafiki, you call the teenagers, put them in groups and try talking to them, hear their views, their comments and try to convince them.”* **Adolescent Youth FGD 3** “*For me, maybe we can use internet because most of the youths are on internet, maybe online. So at least they put information on websites and at least one will have some clue of this and this and that”* **Adolescent Youth FGD 3** “*I think also the government and the health system in general needs to do like a serious campaign just the way they have been doing with the COVID vaccine drugs. Like they need to do mass sensitization of people and run campaigns because you see not so many people are aware of the development of injectable ART and even the injectable PrEP. You see there are some youths who are coming from deep down there in Kakamega, deep down there in Navakholo Sub County, they don't have this information. They are not online and the government also needs to invest in sensitizing and running campaigns on the same.”* **Health Advocate FGD 7** “*I actually have the same feeling that because it is a new product that we are bringing in, before integrating it first let us start by ensuring that they place it in CCC, the people living with HIV, the young people living with HIV are well equipped with information and I am not sure whether it is going to be an optional thing where maybe I can start by injectable and maybe if I feel like it is not good for me I go back to the orals. I am not sure if that is an option but in case that is an option, I feel like at the CCC it is going to be a better place to handle such kind of a situation unlike putting it together with the FP. So, while we are rolling it out and then now people are getting used to it, then they can now move to the integration part…”* **Health Advocate FGD 7**
	Interpersonal level Health	*Family support barriers*	“*Let us say as we had already said-getting permission from school, number two, does the parent have that money to actually bring that adolescent to the clinic every specific day?”* **Healthcare Provider FGD 1** “*So it will also be a burden to the caregivers if they have to accompany their children to the facilities”* **Healthcare Provider FGD 2**
		*Non-adherence due to school logistics*	“*For me, there will be a lot of non-adherence. Apart from being good, you realize that sometimes we have children who are studying in Mombasa or the long distances travelers. So they will not be able to come monthly. And then again you cannot give an injectable to go home with it to be injected. So as much as adherence can improve, it can also be wanting. It can give someone a long term solution.”* **Healthcare Provider FGD 1** “*I concur with number 9 although at the same time I am still imagining stigma has reduced in schools, the issue of carrying drugs to school has reduced but at the same time this is a child who is supposed to be coming to the clinic monthly and then with Magoha's schedule, imagine you are in half term today then in 2 weeks' time again your parent is going back to school to say that the child again has to come to the clinic. The following month again. Still there will be so much questions like every month you are going to the clinic. What are you going to do and you are just from half term. So imagine such kind of questions again.”* **Healthcare Provider FGD 1** “*But the bad news is for instance those who are going to school. That is where my concern is. Basically someone has been taking their drugs well but because of school and maybe they have not disclosed in school, there are those challenges of seeking permission to come to clinic because it becomes tricky in school. So those are some of the concerns that brings negative aspects. But then on the positive side, according to me it has more positives that negative, it is only to find a liberal way of rolling out to these adolescents especially the school going adolescents who are in colleges and those who are in towns. So such consideration needs to be taken keen on so that one will not miss their doses.”* **Adolescent Youth FGD 3**
		*Negative Peer influence*	“*You know adolescents usually talk with each other. They don't talk with the clinician. ‘I used this and I had a head ache, I took this and I had diarrhea'. I remember there is a time an adolescent refused to get to second line because he had heard that he was going to diarrhea and vomit for the next 6 months. They talk among themselves and when the clinician said I am going to initiate you to second line, he refused; ‘I am not going to second line, I am not ready to diarrhea for the next 6 months'. But as soon as the information was disseminated what second line really means, the acceptability was okay.”* **Healthcare Provider FGD 2**
	Health Systems/Institutional level	*Increased provider workload strain*	“*We will be seeing these clients monthly. Considering again the staff-client ration, then we will be overwhelmed because we will have to review them monthly. If we have 800 patients monthly, every month 800 patients coming for monthly injections, I can just imagine.”* **Healthcare Provider FGD 1** “*Now it will also be overwhelming to the clinic within these months that maybe 800 adolescents, you can imagine 800 adolescents are being seen every month, in the next month another 800. It will be overwork for some of the staffs unless we also consider some of those things before it is initiated. Even if it is going to be initiated, let them be initiated in phases like a pilot and then you find the reactions.”* **Healthcare Provider FGD 1** “*Okay. I am just thinking in terms of if people are coming monthly, so say 800 divide by 30 days, we have I don't know like how many. So in a day say you have 50 people. That is 50 people you are injecting. That means we have a nurse who only sits there in the injection room and another nurse now doing the rest of the things now her main work. Given a facility like ours, we do email-clearing and forwarding. There are many things that adolescents undergo, there are many things that adolescents have. Sometime you can spend like a whole hour with one adolescent. So I am thinking if you have to give quality care, you will really need to have more people coming in to support this work”* **Healthcare Provider FGD 1***1 “For me, I am looking in terms of monthly injection. Definitely we will need increased workload in terms of a personnel who will be doing the injection. So it will also increase the workload because it is a monthly injection.”* **Healthcare Provider FGD 2**
		*Dislike of monthly injection regime*	“*Yeah. They can do research and come up with one that can be injected every 3 months or 6 months. That will be okay. But for 1 month, it is going to be a disaster.”* **Healthcare Provider FGD 1** “*It is a good thing but at the same time as number 2 has said if they can consider a longer one, maybe we can have both-monthly, 3 months and 6 months and 1 year. It can be something good.”* **Healthcare Provider FGD 1** “*Number 4 has already talked about because monthly is hard because you can consider that parent who doesn't have a means of transport because we have those ones coming from Kisumu. Siaya, you see. So it will be hard for them to come. If it is 3 months, 6 months, it will be better.”* **Healthcare Provider FGD 1** “*These adolescents-15–24 are expecting an injectable that will last them 6 months or more. It will be a disappointment for them from my point of view to realize that it is something that will be done monthly. First of all they are looking at [Unintelligible 01:26:46] not visiting the clinic all the time all the time, they are looking at coming to the clinic once or twice in a year. So if it comes to a point whereby they will be told this injection will be done every month, they will still not be ready to go with it.”* **Healthcare Provider FGD 1**
		*Storage, Supply, Waste capacity*	“*How will it be stored? Do we have the capacity to store it?”* **Healthcare Provider FGD 1** “*I mean, is it kept in a fridge? And if it is, how prepared are we to have like many fridges?”* **Healthcare Provider FGD 1** “*I would like to inquire maybe these injectable, and do they need conditions like do they need to be refrigerated? To be put in a freezer? If yes, some facilities might be lacking these freezers for them to keep this injectable drug.”* **Health Advocate FGD 7** “*Supply and demand. Supply and demand in the sense that initiating the long acting medication. You remember something like DTG, it was rolled out to so many people ate the same time and then there was a stock out for a period of time.”* **Healthcare Provider FGD 1** “*There will be a lot of waste disposal in terms on needles, in terms of gloves that is involved. So it will be a little bit expensive unlike the pills that they were taking.”* **Healthcare Provider FGD 2** “*The only fear I see with the roll out of the injection is stock. You remember there is a time ARVs were out of stock and you could have some people getting pills for 2 weeks. So you can imagine you have rolled it all at once to all the people living with HIV in Kenya and then the stocks have reduced. So how will one get the dosage? That is where number 5′s point comes through where you are given a short dose that will push you for a month or half a month or you will not get an injection completely. So that is the only fear that I see in terms of rolling out. It should be rolled out in phases so that at least there will be that consistency of supply*.” **Adolescent Youth FGD 3** “*Even in terms of personnel, the clinics also have been having task shifting. We rely on CCC assistants, we have been relying on retention peers to support the clinic but now you realize the injection will require either a nurse or a clinician to give and that might be overwhelming to the clinic and also the supplies. We need the syringe, we need the needles. Are we ready for that? I don't think so”*. **Healthcare Provider FGD 2**
		*Need for trained staff*	“*About the human resource, we have that nurse for example in Rafiki at the moment, it will mean the rest of us will be trained on giving those jabs. So I am thinking about the staff, I am thinking about the storage, I am thinking about umm…as we heard the monthly thing, the huge number of patients. How possible will that be?”* **Healthcare Provider FGD 1** “*You train the health workers and convince them that it is workable because they are the ones who will disseminate this information to clients. If the health worker is not convinced that this injectable is working, there is no way that roll out will be successful.”* **Healthcare Provider FGD 2**
		*Need for infection prevention*	“*at the same time the infection prevention will really have to be upped as opposed to oral medication which we are really not actively doing infection prevention.”* **Healthcare Provider FGD 2**
		*Conflict with differentiated service delivery model*	“*It will be like rolling back the gains of differentiated service delivery whereby people are given a chance to go for 6 months or even one yeah without visiting the clinic. Now they will be required to come every month. And it will inconvenience them.”* **Healthcare Provider FGD 2**
		*Logistics and scheduling*	“*For me what I can propose, the first thing is about resistance. What we need to look at is the logistics on the schedules that registration of these medicine. What are the schedules? What are the logistics and what are the acceptable variances for administration? These have to be timed to the school programs. If I am umm…. mostly likely I have the university girls because people from 20, twenty something or 19, they are all could be in the university. Others in high schools or technical colleges. We need to look at the logistics and the first thing is acceptable variance of in terms of administration, how many days am I allowed to be within?”* **Policy Maker FGD 6** *One of the major shifts that is going to affect how the programing around HIV has been over the years is the issue of this injectable needing to be administered by a health care provider. As (Name) has said, most of our clinic setups would need to have a bit of reorganization for us to allow an interface of referral for injection and really the dependence on qualified health workers to administer the drug then becomes something that we consider as a shift of planning.”* **Policymaker FGD 6** “*there are facilities that are running Comprehensive Care Centers (CCC) with very minimum staff levels and therefore additional requirements for more intense health worker intervention would require for us to review the capacities of these facilities for them to meet the load of clients on a monthly basis because some of these clients because of how we have reorganized our clinics they come on a quarterly basis or they come every six months. In terms of then the cost and what has been discussed, we would consider the cost implications into the public health programs, how many people we will be able to cover with the resources we have when we introduce this”*. **Policymaker FGD 6**
		*Pharmacy Protocols; combined treatment with LA ART*	“*Not really. I don't know whether I can be allowed to ask a question now that we have dealt with the long acting and you have done the studies. Is there a way that you can use that monthly injection and at the same time use the orals? Maybe we can consider now that maybe I am not available the next month, you can do the one month injection and once the one month is finished you can go to the orals. Is it possible?”* **Healthcare Provider FGD 1** “*There is the issue of drugs interaction, what it means combining with family planning especially among the adolescents population, issues around acceptability among these populations is something that we would also consider as we do the guideline, decisions and considerations for the product as we roll out”* **Policy Maker FGD 3** “*What are the other factors to consider apart from the interruption of these medicine with contraceptives? Be they oral or whichever type of contraceptives and I believe you are focusing more on umm…I will not talk about that because I need to go back and see what you are focusing on. The next thing also is, would you want to include it as a secondary objective if there are other medicines that could have potential interruption? Would you want to include them? That is another thing. As we know the TB medications, some of the TB medications like - [Unintelligible 00:44:39] would you want to consider? Do they have an impact? Do they have an impact on efficacy?”* **Policy Maker FGD 6** “*So maybe to the adults, questions like maybe you are suffering, you have blood pressure, diabetes and all others, you know-how will it affect them? Sor the adults there are other complications that come along with it.”* **Health Advocate FGD 5**
		*Cost-effectiveness and Sustainability*	“*Usually one of the things that we consider is cost effectiveness, acceptability of the product and sustainability of new entries as we start given this is a public health program, and that we would take into consideration as we consider the new forms of long acting injectable antiretroviral.”* **Policy Maker FGD 3** “*I also had a question about what is the cost implication when we want to scale up this and how sustainable to have this over a long….”* **Policy Maker FGD 3**

### Part 1: benefits to LA ART

The analysis below explores the potential benefits of LA ART for AYPLHIV. The study participants identified significant advantages across individual, interpersonal, and health system levels (noting no policy-level benefits arose from our data). Notably, individual-level benefits emerged as particularly promising, with AYPLHIV highlighting LA ART's potential to improve adherence, reduce stigma, and enhance mental wellbeing.

#### Individual

Participants across all groups identified numerous individual-level benefits associated with LA ART, viewing it as a promising approach to improve adherence, reduce stigma and pill burden, aid in privacy, and potentially improve mental wellbeing for AYPLHIV. These interrelated, and at times overlapping, themes will be further detailed below.

##### Improved ART adherence

LA ART was perceived as a promising strategy to address adherence challenges, particularly among AYPLHIV. Healthcare providers emphasized how monthly injections could simplify medication routines and reduce pill burden. This, they believed, would lead to improved adherence and potentially decrease stigma associated with taking daily pills, particularly when at school. AYPLHIV echoed these sentiments, highlighting the challenges of managing oral ART discreetly in school settings. They viewed LA ART as a way to avoid disclosure and maintain treatment continuity even during social events. Both healthcare providers and AYPLHIV also mentioned the benefit of reduced missed doses associated with less frequent injections compared to daily pills. Overall, LA ART was seen as a potential solution for promoting adherence and addressing social barriers to treatment, particularly for AYPLHIV.

“*It is good because you will not keep on taking the drugs. You will be 100% sure that you are in LDL [lower limit of detection, a laboratory term connoting undetectable for HIV viral load]. Like you see sometimes, if you have been taking your drugs at 7 and then maybe you had gone somewhere and you come back late, and when you go and take your LDL, you will not be sure you are on LDL, but with injection you are sure you are on LDL.”* **Adolescent Youth FGD 4**

##### Reduced HIV stigma, inadvertent disclosure, and improved privacy

AYPLHIV consistently identified reduced stigma, inadvertent disclosure, and maintenance of privacy as key benefits of LA ART. Disclosing their HIV status and managing daily medication in social settings were major concerns. The daily routine of taking pills was seen as a challenge, potentially leading to awkward situations with friends and difficulty maintaining discretion, especially in boarding schools. Even providers acknowledged the risk of inadvertent disclosure from pill bottles rattling in a bag (“kayamba” refers to the noise of pills rattling in a bottle in Kiswahili), particularly when people were traveling which AYPLHIV do frequently due to attending boarding schools. They felt LA ART's injections would offer greater privacy as there would not be a need to take a medication in front of friends, peers, or family. Participants stated that monthly injections offered a solution by eliminating the need for visible medication and reducing opportunities for questions or suspicion from peers. This potential for increased privacy was viewed as a way to manage stigma and avoid negative social interactions associated with HIV. LA ART would potentially improve AYPLHIV' confidence and freedom within social settings, allowing them to participate in activities, such as sleepovers, without worry. Additionally, from a patient-centered perspective, policymakers recognized that the long-term benefits of LA ART could be extended by the possibility of self-administration to improve convenience for patients and reduce burden on health systems.

“*And then again you can also travel like she has said. You can go for sleepovers [laughing…] like you see it is difficult for me to leave my home and go somewhere because of the medicine. I do not have that privacy in other places.”* **Adolescent Youth FGD 3**

##### Reduced pill burden and increased ART effectiveness

Healthcare providers and AYPLHIV highlighted challenges with carrying medication while traveling. Providers noted concerns about medications losing potency due to improper storage, and the difficulty of maintaining a daily routine on the go. They also acknowledged “pill fatigue” as a reason for missed doses, comparing it to people not finishing courses of medication like malaria tablets. AYPLHIV echoed these sentiments, emphasizing the convenience of LA ART's reduced dosing schedule. They saw monthly injections as a way to avoid the burden of daily medication routines and the potential for forgetting pills during busy days at boarding school, work, or travel. LA ART's extended-release formulation would address pill burden and ultimately adherence challenges associated with busy lifestyles and travel, leading to greater effectiveness of ART among and overall improved treatment outcomes for AYPLHIV.

“*You know going to parties or going to travel, so carrying medications sometimes loses its potency because walking around with the bottle, we call it ‘kayamba' when you put it in the bag it is like ‘chaka- chaka- chaka- chaka' so it will be easier for those people who travel a lot*.” **Healthcare Provider FGD 1**

##### Improved mental health

AYPLHIV discussed potential reductions in stress and depression associated with living with HIV and taking oral ART that LA ART could help alleviate. They suggested that the less frequent dosing schedule (e.g., every monthly injections) could alleviate the constant reminder of their HIV status that might accompany daily pill routines. This shift in mentality, from taking daily medication to going to the clinic for a single, monthly injection, was likened to receiving a salary—a source of relief and a prompt to focus on other aspects of life, potentially improving overall mental wellbeing and reducing depression.

“*Yeah, so I see the rate of depression will reduce. So, if you look at the statistics, it will reduce that mentality. It will reduce the mental health and depression. You will just be thinking that when it is end of the month, you get that injection, it is like salary and you will be going to get that salary. You get that injection and you will not have that mentality. Your brain will be used to do many other things including your job work, your school work.”* **Adolescent Youth FGD 4**

##### Potential for integration with family planning services

The FGDs revealed the potential benefits of combining family planning services and LA ART to streamline care and reduce the burden on patients, especially among AGYWLHIV. Healthcare providers emphasized that offering both services simultaneously—family planning and HIV care—can simplify access for patients, particularly AYPLHIV who are already attending HIV clinics. An advocate also highlighted the need to address challenges in contraceptive uptake, especially among younger populations. While older women are more likely to use long-acting contraceptives, young girls are often excluded from these discussions, emphasizing the importance of educating them about their options and empowering them to make informed decisions. Additionally, the convenience of long-acting methods—whether for family planning or ART—was noted, with the suggestion that AYPLHIV familiar with the ease of long-acting contraceptives may be more likely to choose LA ART. They highlighted a convergence in use of long-acting methods for both family planning and HIV treatment, possibly even for injectable methods for the two. Overall, AYPLHIV, providers, advocates, and policymakers collectively highlight the importance of integrating services to improve access, promote informed decision-making, and increase uptake among younger populations.

“*I would say that an adolescent already using long-acting family planning could be influenced to opt for the long-acting injectable for ART. If they've been benefiting from just one shot and then not having to worry about keeping track of a schedule, that convenience could encourage them to choose the long-acting injectable for ART as well.”* **Advocate FGD 5**

#### Interpersonal-level

At the interpersonal level, participants, perceived LA ART as a more private ART option, improving their social interactions and relationships.

##### Improved relationships by reducing the risk of inadvertent disclosure

LA ART offers a significant interpersonal benefit for AYPLHIV by promoting greater privacy and reducing the risk of inadvertent disclosure of their HIV status. This can be particularly helpful for AYPLHIV in romantic relationships, as highlighted by a participant who was concerned that their boyfriend may encounter them receiving ART at a clinic. AYPLHIV also emphasized the potential for awkward situations due to the need to carry and manage pills. One participant described the difficulty of leaving a party or social gathering to take medication discreetly. Consequently, LA ART's monthly injections were viewed as a solution by eliminating the need for visible medication and reducing opportunities for suspicion of HIV. This increased privacy allows AYPLHIV to navigate their social interactions with more confidence and fosters stronger interpersonal relationships by reducing the fear of inadvertent disclosure.

“*…the advantage is, you find for example adolescents who are out of school or in campuses and are dating, the problem of keeping your medication where no one can find. You will have eliminated that one. So accidental disclosure among other adolescents. That will be one thing.”* **Healthcare Provider FGD 1**

##### Family support of LA ART, due to reduced burden on them

Parental influence emerged as a factor for successful ART adherence, particularly for younger AYPLHIV who are not yet independent enough to be in charge of their own oral ART intake, so require parents or other family members to administer the medications or remind them to take their medications. So, LA ART would be a welcome relief for the family members too. For example, a participant highlighted the challenges faced by primary school children in managing daily medications independently, and this may be a motivating factor for children to take LA ART. This also highlights the role parents or other family members will likely play in supporting their children's transition to LA ART.

“*I think what will influence those who are in primary school is to take the injection as the guidance from the parent, because obviously they are not independent. So, the parent will influence*.” **Adolescent Youth FGD 3**

##### Positive peer influence

Positive peer influence emerged as a potential benefit for overcoming initial hesitancy toward using LA ART, particularly among AYPLHIV. Discussions amongst AYPLHIV revealed concerns about new medications and potential side effects, similar to their experiences when needing to switch to second-line ART. However, participants envisioned a snowball effect, driven by positive peer experiences, countering these concerns. They suggested that successful early adopters of LA ART could serve as role models, generating positive word-of-mouth messages and attracting more AYPLHIV to the program. This potential for positive reinforcement could counteract initial hesitancy and contribute to increase LA ART uptake, although careful management strategies may be required to address a potential surge in demand exceeding initial enrollment targets providers. Furthermore, one adolescent participant suggested using LA ART as a potential motivator for improved adherence among their peers. They envisioned a scenario where AYPLHIV with undetectable low viral loads would be the only ones eligible for the injectable medication, creating a positive incentive for others to achieve the same undetectable viral load status, i.e., creating a culture to incentive being virally suppressed. By offering LA ART as a reward for achieving viral suppression, AYPLHIV who are struggling with adherence could be motivated by the success of their peers. Seeing others benefit from the convenience and potential advantages of LA ART could encourage them to improve their own adherence behavior.

“*It will be like a motivation thing to the youth because if you give someone with a low viral load, this one will ask; ‘why am I not being given?' the reason behind it is that he/she is not virally suppressed. So, if you tell him/her that if you improve your viral load, you will be able to acquire the injection for example if it is once a month or depending on how the injection will be...”* **Adolescent Youth FGD 3**

#### Health systems/institutional

LA ART offers a broader range of treatment options for patients and providers in places like Kenya, which have not historically had *options* to offer their patients. Discussions amongst providers highlighted limitations of current oral ART choices due to overall resource constraints in LMICs. LA ART, with its extended dosing interval and attraction to so many patients, could become a valuable addition to the first-line ART regimen options. This would provide clinicians with more flexibility in tailoring treatment plans to individual needs, potentially improving overall care and population-level targets for HIV.

“*Case in point, we had an opportunity to have training with a university abroad and we just learnt that the choices of first line for their clients is not really dependent on the guidelines that they have but the choice lies with the clients because they have a variety to choose from. Unlike a country which is categorized as low- or middle-income country, where we have limited choices. So, if we reach such a place where the variety is wider, then I think we will be able to serve our clients better.”* **Healthcare Provider FGD 2**

### Part 2: barriers to LA ART uptake

The successful implementation of LA ART for AYPLHIV requires careful consideration of potential barriers. The study participants identified a range of barriers across the individual, interpersonal, and health system/institutional levels. Notably, no policy-level barriers emerged; instead, health system concerns were identified as the most significant obstacles. Healthcare providers expressed anxieties about increased workloads, potential disruptions to existing service delivery models, and the need for additional training and infrastructure. The analysis below delves deeper into the specific barriers identified within each socioecological model level.

#### Individual level

Several concerns were raised at the individual level regarding the use of LA ART. Participants in the study expressed apprehensions about a lack of knowledge surrounding LA ART and usual concerns of effectiveness associated with new medications.

##### Lack of accurate LA ART information

A significant barrier to LA ART rollout identified by both AYPLHIV and healthcare advocates is the lack of awareness and education. AYPLHIV expressed a general lack of knowledge about LA ART, including details about side effects, dosages, and how to handle variations in the monthly vs. 2-monthly dosing schedules. This knowledge gap can lead to hesitancy and distrust, potentially fueled by existing misinformation. Various types of participants thought it was best to address this knowledge gap preemptively, before rollout of LA ART actually starts. To combat misinformation and promote informed decision-making, AYPLHIV suggested various communication strategies like group discussions, online platforms, and mass campaigns similar to COVID-19 vaccine initiatives. Reaching AYPLHIV in remote areas requires additional efforts beyond solely relying on online resources. Effective communication and education campaigns, using a multi-pronged approach with tailored messages for different segments of the adolescent population, will be crucial to overcome this barrier.

“*For me, maybe we can use internet because most of the youths are on internet, maybe online. So at least they put information on websites and at least one will have some clue of this and this and that”* **Adolescent Youth FGD 3**

##### Fear of needles, side effects, and effectiveness of new drugs

The transition to LA ART might be hindered by a fear of needles among some AYPLHIV. Healthcare providers acknowledged this concern, particularly for younger children who may not tolerate injections well. AYPLHIV themselves expressed anxieties about the pain associated with injections and the potential disruption the pain might cause to their daily routines. Furthermore, some AYPLHIV and young children may strongly prefer oral medication over injections, underscoring the importance of providing multiple treatment options to accommodate personal preferences and avoid adherence challenges. Additionally, uncertainties surrounding potential side effects emerged as another individual-level barrier. These concerns ranged from the possibility of severe reactions to injections and to how LA ART might interact with their bodies, mirroring anxieties observed during the COVID-19 vaccine rollout. Other AYPLHIV expressed apprehension about a new medication and its effectiveness. AYPLHIV were concerned that the medication dosage might not be enough to last the entire month, potentially leading to a rise in viral load and subsequent illness. These fears highlight the need for effective communication strategies to emphasize the safety and effectiveness of LA ART, addressing concerns about fear of needles and pre-emptive messaging for potential side effects and mechanisms for maintain its effectiveness.

“*Yes, the fear of accepting it. You don't know, has it ever been tested on anyone else? That is the first thing I will ask for me. This is because maybe my body will react very differently. Maybe I will become sick. So, I will need to know first if it has been tested on anyone.”* **Adolescent Youth FGD 3**

“*Circumstances come in especially for the children and those people who fear the injection. There are those people who do not go one way with injections. They prefer orals. That is where point number one comes through so that it becomes a choice to those who don't prefer injections, they can just use the pills. And then definitely you know the young children don't get along with injections. So I am feeling it will still be a challenge to them”* **Adolescent Youth FGD 3**

##### Dislike for increased clinic visits

While current LA ART offers a monthly or two-monthly injections, some patients expressed a preference for the existing system of receiving oral ART every 3–6 months due to fewer overall clinic visits. Of note, while Kenyan guidelines generally recommend monthly follow-up for children living with HIV, AYPLHIV often receive fewer visits, either due to entering differentiated service delivery models that space out their visits or inability to return to clinic more frequently due to boarding school or other school-related reasons.

“*I also agree because when the patients ask about the injectable, they thought it will take around 3–6 months then they come for another injection. So when I told them it is monthly, they said ‘I would rather continue with my pill”'* **Healthcare Provider FGD 2**

##### Added complexity of LA ART for missed doses

While most participants overwhelmingly appreciated the benefits of LA ART, some expressed concerns about the added complexity of missing LA ART doses and having to bridge with oral ART. This complexity is particularly relevant to AYPLHIV given their inclination for overall risk-taking behaviors. One adolescent highlighted how experimental tendencies could lead to manipulation of the treatment regimen. For example, if an adolescent misses a monthly LA ART injection, they might attempt to compensate by taking oral ART for a few days and then falsely claim during their clinic visit that they had not taken any oral medication at all, simultaneously trying to prevent viremia but also being afraid to acknowledge to a provider of their non-adherence to LA ART injection visits. This situation underscores the necessity of implementing adherence support strategies tailored to the unique needs and potential challenges faced by AYPLHIV. By addressing these complexities, healthcare providers can better support young patients in maintaining their treatment regimens and achieving optimal health outcomes.

“*For us adolescents we really like to explore. The way number 4 has said, I will be using a jab and I will be thinking; what if I use…if I have missed a jab for 2 days and so let me take 2 days' tablets and then when I go to clinic I will say I didn't take any medication. So that is the kind of challenges we will get.”* **Adolescent Youth FGD 3**

#### Interpersonal level

AYPLHIV face a range of interpersonal barriers that hinder their ability to consistently adhere to LA ART regimens. Key challenges include logistical burdens on caregivers, school-related conflicts, and the influence of misinformation from peers, all of which can significantly impact treatment adherence and access to care.

##### Family support barriers

Healthcare providers identified logistical concerns for AYPLHIV and their caregivers. One concern expressed by providers was the financial and time burden on caregivers who need to accompany their children, including younger adolescents, to more frequent clinic appointments. If the AYPLHIV currently have less frequent clinic visits with oral ART, e.g., every 3–6 months, then more frequent visits to get the LA ART, even if not with their providers but with other clinic staff to simply get their injections, still engenders an added financial and time burden for the patients.

“*So it will also be a burden to the caregivers if they have to accompany their children to the facilities.”* **Healthcare Provider FGD 2**

##### Non-adherence due to school logistics

Healthcare providers and AYPLHIV themselves raised concerns about the feasibility of monthly clinic visits for LA ART. Logistical hurdles like long travel distances from school or tight academic schedules could make it difficult for AYPLHIV to attend appointments consistently. Most importantly, both groups expressed anxieties about potential non-adherence to injection visits. Frequent clinic visits now for monthly injections might raise questions from peers and school staff, especially for AYPLHIV who have not disclosed their HIV status. These barriers highlight the need for flexible scheduling options, potential school-based administration systems, and exploring alternative approaches like self-injectables that could reduce the frequency of clinic visits while maintaining adherence.

“*For me, there will be a lot of non-adherence. Apart from being good, you realize that sometimes we have children who are studying in Mombasa or the long distances travelers. So, they will not be able to come monthly. And then again you cannot give an injectable to go home with it to be injected. So, as much as adherence can improve, it can also be wanting. It can give someone a long-term solution.”* **Healthcare Provider FGD 1**

##### Negative peer influence

Misinformation from peers emerged as a significant interpersonal barrier to treatment adherence. Healthcare providers observed that AYPLHIV often rely on information shared amongst themselves regarding medications, rather than consulting with healthcare professionals or other adults. This can be problematic, as inaccurate rumors can lead to treatment refusal. One provider highlighted a specific instance where an adolescent refused to switch to a second-line regimen based on rumors of severe side effects shared by peers. This incident underscores the potential for negative peer influence to create treatment hesitancy based on misinformation, potentially posing a barrier to LA ART uptake. Effective communication strategies are crucial to counteract this challenge, such as ensuring AYPLHIV have access to accurate information about LA ART and thoughtfully showcasing early adopters, to mitigate the influence of potentially misleading peer information.

“*You know adolescents usually talk with each other. They don't talk with the clinician. ‘I used this and I had a head ache, I took this and I had diarrhea.' I remember there is a time an adolescent refused to get to second line because he had heard that he was going to have diarrhea and vomit for the next 6 months. They talk among themselves and when the clinician said I am going to initiate you to second line, he refused; ‘I am not going to second line, I am not ready to have diarrhea for the next 6 months.' But as soon as the information was disseminated what second line really means, the acceptability was okay.”* **Healthcare Provider FGD 2**

#### Health systems/institutional level

As healthcare providers prepare for the rollout of LA ART, a major concern is the potential strain on the healthcare system due to increased workloads. Providers expressed apprehension about managing a higher volume of patients receiving monthly or 2-monthly injections, particularly in light of already high patient-to-staff ratios. This section explores the logistical challenges associated with administering LA ART, including the impact on existing service delivery models and need for additional training and dedicated personnel to handle injections. Addressing these concerns is crucial to ensure that healthcare providers are equipped to deliver effective care while optimizing the benefits of LA ART implementation.

##### Increased provider workload strain and training

A major concern raised by healthcare providers was the potential strain on the healthcare system due to increased workload with LA ART. The prospect of seeing a higher volume of patients monthly or 2-monthly, potentially hundreds at a time, was daunting considering existing high patient-to-staff ratios. One provider worried about the logistics of managing monthly injections for such a large group, potentially requiring dedicated personnel just for injections. This could significantly increase the overall workload for healthcare staff who already handle various tasks beyond medication administration. These concerns highlight the importance of finding solutions to address potential staffing shortages preemptively to optimize the benefits of LA ART rollout. This might involve implementing LA ART in phases, providing additional staff training, or exploring alternative models for workload distribution. Additional concerns were raised about the capacity to administer injections, suggesting a need for training existing staff on proper injection techniques and a heightened focus on infection prevention protocols compared to oral ART. Healthcare providers emphasized the importance of equipping staff with the knowledge and confidence to effectively communicate the benefits of LA ART to AYPLHIV. Addressing these staffing and training needs will be crucial for optimal patient care and a smooth LA ART implementation.

“*Now it will also be overwhelming to the clinic within these months that maybe 800 adolescents, you can imagine 800 adolescents are being seen every month, in the next month another 800. It will be overwork for some of the staffs unless we also consider some of those things before it is initiated. Even if it is going to be initiated, let them be initiated in phases like a pilot and then you find the reactions.”* **Healthcare Provider FGD 1**

##### Conflict with differentiated service delivery model, disklike of monthly injection regime, logistics, and scheduling

Healthcare providers expressed concerns about the practicality of monthly injections for LA ART. A significant barrier is the potential disruption to the existing differentiated service delivery models, which allows patients doing well to collect medication refills for extended periods, at times up to 12 months, minimizing clinic visits. This established system could be disrupted by the need for monthly injections, potentially inconveniencing AYPLHIV. Further complicating matters are scheduling difficulties around school programs. A policymaker highlighted the need for flexible scheduling options and acceptable variances in administration times to accommodate these academic commitments. Several providers suggested exploring alternative solutions beyond monthly injections. Options like 3-month, 6-month, or even year-long intervals were preferred. These longer intervals could benefit AYPLHIV facing transportation challenges or who desire less frequent clinic visits. Regardless of the injection frequency, ensuring convenient access and flexible scheduling will be crucial for adolescent adherence to LA ART. This may require exploring alternative models and working with schools to minimize disruption to academic schedules. Healthcare providers also expressed concern over logistics of offering oral ART bridging options with LA ART injections. While the oral bridging approach could address concerns about missed injections due to travel limitations or other scheduling conflicts, it added layers of logistical concerns in forecasting demand for the oral bridge options.

“*It will be like rolling back the gains of differentiated service delivery whereby people are given a chance to go for 6 months or even one yeah without visiting the clinic. Now they will be required to come every month. And it will inconvenience them.”* **Healthcare Provider FGD 2**

##### Storage, supply chain, and waste capacity

Beyond logistical challenges with patient visits, healthcare providers and AYPLHIV identified significant barriers related to storage, supply chain, and waste disposal that could impact LA ART rollout and health system readiness. Concerns regarding storage centered on the availability of appropriate facilities, particularly refrigerators, to maintain the medication at the required temperatures. Maintaining the cold supply chain of LA ART was seen as another issue. Additionally, past experiences with stockouts of other medications fueled anxieties about potential supply chain disruptions for LA ART. Phased implementation was suggested as a potential solution to ensure a consistent flow of medication and avoid treatment interruptions. Finally, the increased waste generated by needles, syringes, and gloves associated with injections raised concerns about environmental impact and cost-effectiveness compared to oral ART. Addressing these storage, supply chain, and waste management challenges will be crucial for a successful LA ART rollout and ensuring health systems are adequately prepared to meet the demands of this new treatment approach.

“*I would like to inquire maybe these injectable, and do they need conditions like do they need to be refrigerated? To be put in a freezer? If yes, some facilities might be lacking these freezers for them to keep this injectable drug.”* **Health Advocate FGD 7**

“*Supply and demand. Supply and demand in the sense that initiating the long-acting medication. You remember something like DTG, it was rolled out to so many people ate them at same time and then there was a stock out for a period of time.”* **Healthcare Provider FGD 1**

**“***The only fear I see with the roll out of the injection is stock. You remember there is a time ARVs were out of stock and you could have some people getting pills for 2 weeks. So you can imagine you have rolled it all at once to all the people living with HIV in Kenya and then the stocks have reduced. So how will one get the dosage?”* **Adolescent Youth FGD 3**

##### Cost-effectiveness and sustainability

A significant health system barrier identified by policymakers is the uncertainty surrounding the cost-effectiveness and sustainability of the LA ART rollout. While the potential benefits of LA ART are recognized, policymakers emphasize the need to assess its cost implications in the context of a public health program. They highlight the importance of ensuring the long-term sustainability of this new treatment approach to guarantee its continued availability for those who are transitioned to LA ART. Addressing these concerns was highlighted to require careful cost analyses and exploring sustainable funding models to ensure LA ART remains accessible within the Kenyan healthcare system. Policymakers identified several factors requiring further exploration before implementation, such as potential drug interactions with family planning methods and TB treatments. These considerations highlight the need for thorough treatment policy development and further research to ensure the safe and effective integration of LA ART into existing treatment plans.

“*Usually one of the things that we consider is cost effectiveness, acceptability of the product and sustainability of new entries as we start given this is a public health program, and that we would take into consideration as we consider the new forms of long acting injectable antiretroviral.”* **Policy Maker FGD 3**

“*I also had a question about what is the cost implication when we want to scale up this and how sustainable to have this over a long….”* **Policy Maker FGD 3**

## Discussion

The discussion on health system readiness and service integration for LA ART reveals both optimism and concerns among AYPLHIV, healthcare advocates, providers, and policymakers. AYPLHIV expressed enthusiasm for LA ART's potential to provide convenience, privacy, and freedom, improving adherence and mental health while reducing stigma associated with oral ART, particularly for those in boarding schools, traveling, or dating. Furthermore, there were points of convergence between LA ART and family planning use that could be helpful at the individual and health systems levels, an element particularly salient to AGYWLHIV. However, they highlighted challenges such as long wait times, inconsistent medication access, and confidentiality concerns in clinic settings where HIV care is not fully integrated. Healthcare providers echoed these concerns, pointing to inadequate infrastructure and insufficient staff training for managing LA ART, with many clinics lacking capacity for large-scale injection administration. They raised issues with LA ART's compatibility with the extended return visits established by differentiated service delivery models in LMICs and cited financial implications for more frequent patient visits. Policymakers emphasized the need to address structural and policy barriers for successful LA ART integration, advocating for national guidelines, resource allocation, and robust supply chain and waste management systems to ensure sustainability and cost-effectiveness. They stressed that addressing these systemic factors and developing targeted LA ART messaging would be critical for successful implementation in LMICs. Results from this study highlight the multifaceted facilitators and barriers to LA ART adoption, underscoring the importance of optimizing healthcare systems and preparing comprehensive information ahead of rollout to support comprehensive, adolescent-centered service models.

The benefits indentified in this study align with existing research highlighting LA ART's potential to improve HIV treatment for AYPLHIV. LA ART can improve adherence to treatment regimens, leading to better health outcomes and potentially reducing the stigma associated with daily medications (14, 15). Studies in HICs have shown high satisfaction levels with LA ART, with participants reporting better experiences, despite injection site reactions, and overall satisfaction compared to prior oral ART (33). Furthermore, studies conducted in LMICs, including surveys in the Dominican Republic and Tanzania, suggest that individuals are more likely to choose 3-month LA ART injections over daily oral ART, particularly when access to clinics is challenging (12). An additional dimension of saliency for LMICs regarding LA ART is its compatibility with co-administration of long-acting contraceptives. This combined approach could enhance the feasibility and appeal of LA ART, as it addresses both HIV treatment and family planning needs in a single visit, reducing the frequency of healthcare appointments. This is particularly advantageous for AGYW or adolescents in remote regions with limited access to healthcare facilities, as it could improve adherence, streamline care, and contribute to better health outcomes. Overall, this highlights the potential of LA ART to improve adherence and health outcomes especially for AYPLHIV living in remote regions with limited access to healthcare facilities.

Despite the promising outlook for LA ART in AYPLHIV, our study identified challenges related to health system preparedness, such as increased patient volume, staffing shortages, and increased costs, that mirror experiences with limited LA ART rollout in HICs (34). These challenges highlight the importance of a thoughtful approach in LMIC settings to avoid missteps encountered elsewhere. As documented in HICs, factors like increased patient volume due to more frequent clinic visits for injections (every 2 months compared to potentially up to annual visits for oral ART) could overwhelm existing clinic capacity (34). A streamlined appointment system with reminders and designated clinic sessions, alongside walk-in options for missed appointments, will be crucial to manage this. Learning from HIC experiences, where patients expressed a preference for less frequent injections (every 6 months or annually) suggests the need for exploring longer duration options, self-injection, or pharmacy-administered options in the future to reduce patient burden (34). In LMICs, differentiated service delivery has successfully operated for nearly a decade to alleviate the burden of HIV care on overburdened health systems, making the introduction of even a 2-monthly injection option counterproductive to this momentum (35, 36). Additionally, staffing shortages in LMICs could be exacerbated by the need for trained personnel to administer injections (13). Optimizing appointment scheduling and increasing hiring of qualified healthcare providers, such as nurses and pharmacists, could help mitigate this challenge (13). Increased patient volume may also necessitate expanding existing clinics or extending hours. In the long run, exploring alternative dispensing sites, such as community pharmacies, or utilizing community health workers could alleviate pressure on traditional HIV care systems. However, utilizing a cadre of community health workers to administer injections within the community could enhance accessibility; for instance, community health workers, who have historically provided injectable family planning options in the community (37–39), could provide regular injections at boarding schools, thereby minimizing the need for AYPLHIV to travel. By proactively building in such programs ahead of the LA ART rollout, LMIC health systems can ensure a more effective integration of this treatment into existing care frameworks.

Integrating LA ART in LMICs necessitates careful consideration of logistical challenges, including financial constraints, cold chain storage requirements, and waste management, to ensure effective implementation and maintain the drug's efficacy. Firstly, financial considerations warrant significant attention regarding planning rollout of LA ART in LMICs. A cost-effectiveness analysis indicates that injectable LA ART must be priced at no more than $131 USD per year to be considered cost-effective for patients who are not fully virally suppressed, while standard oral ART costs $78 USD (40). Currently, the list price for injectable therapies in the U.S. exceeds $48,000 USD per year for injectable cabotegravir/rilpivirine, though these prices may decrease as discounts are negotiated (43, 44). Secondly, storage and staffing are critical considerations for integrating LA ART in LMICs. Cabotegravir/rilpivirine requires cold chain storage, necessitating upgrades in refrigeration and electricity capacity for some clinics, which poses significant barriers to maintaining the drug's efficacy in warmer climates (41). Thirdly, the increased waste generated by needles, syringes, and gloves associated with LA ART injections raises concerns about sustainable waste management, especially in LMICs where waste disposal systems are often underdeveloped. In many African countries, the lack of national guidelines for medical waste disposal exacerbates the problem, leading to improper handling and storage of waste in healthcare facilities (42). Incineration, while commonly used for its efficiency in reducing waste volume, can create hazardous environmental risks if not done with the right technology (42). To address these challenges, it is crucial to implement sustainable, eco-friendly waste disposal solutions, ensuring both environmental protection and the resilience of healthcare systems in LMICs.

Moreover, the experience of HICs demonstrates that LA ART implementation is achievable despite initial challenges. A study in the U.S. found that barriers decreased significantly over time, with most healthcare staff believing optimal implementation took 1 to 3 months (22, 43). By proactively addressing these challenges through thoughtful planning, learning from HIC experiences, and employing effective communication, LMICs can pave the way for a successful LA ART rollout that improves the lives of AGYWHLGIV.

Finally, adherence challenges can differ significantly between AYPLHIV with perinatally acquired HIV, who have been on ART since early childhood, and those with behaviorally acquired HIV, who often initiate ART later in adolescence (44, 45). While our study did not explicitly sample based on mode of HIV acquisition, and thus we cannot confirm the distribution of perinatally vs. behaviorally infected participants, it is likely that many participants under 18 years of age who had been on ART for 11+ years were perinatally infected. Perinatally infected youth may experience treatment fatigue, as they have been living with HIV for a longer period, leading to potential challenges in long-term adherence (45). Conversely, behaviorally infected youth may face unique stigma and disclosure concerns related to their HIV status, which can influence their adherence behaviors (45). This is in line with a study of AGWYLHIV, which found that while perinatally and behaviorally infected individuals had similar knowledge of reproductive health and family planning practices, behaviorally infected youth reported higher levels of depression and were more likely to have been pregnant (45). Despite adequate health knowledge in both groups, behaviorally infected youth had lower adherence to contraceptive methods (45), which may reflect broader adherence issues with ART. These findings underscore the need for tailored support strategies for each group, addressing both the psychosocial factors that affect treatment adherence and the specific challenges posed by their infection histories.

### Limitations

While this study provides valuable insights into the perceived benefits and barriers of LA ART among AYPLHIV in Kenya, its limitations should be considered in interpretation of the findings. First, our work focused on the perspectives of AYPLHIV, healthcare providers, advocates, and policymakers. Including other stakeholders, such as parents or guardians, in future studies could capture a more comprehensive view of the benefits and barriers of LA ART implementation. Second, the study design itself presents limitations that could affect the transferability of the findings. The study clinic we recruited from is very specialized and unique, offering youth-friendly services in an optimized manner. Most HIV care for AYPLHIV falls far short of this level of integration. Therefore, the insights and past experiences of the AYPLHIV and health providers at this clinic may be highly biased and not easily transferable to other settings within Kenya or other LMICs. Additionally, our study lacks capturing the mode of HIV acquistion, potentially resulting in the underrepresentation of adolescent girls with behaviorally acquired HIV, may limit the generalizability of our findings and the ability to fully capture the potentially significant differences between adolescents with perinatally and behaviorally acquired HIV. Finally, the policy makers we recruited were largely persons working at the programmatic, county level rather than the national level. Consequently, their views are likely aligned to specific programmatic barriers may not be directly applicable to national guideline-making. Moreover, we only conducted one FGD with policy makers, as we could not identify sufficient potential participants for a second FGD. Despite this, the data collected from the policymakers provided valuable insights into structural and policy barriers to LA ART implementation. Despite these limitations, this study serves as an important initial investigation into the perceptions and experiences surrounding LA ART among AYPLHIV in Kenya. Future research endeavors should address these limitations to build upon and refine our understanding of the complexities associated with the implementation of LA ART in diverse healthcare settings.

## Conclusion

LA ART is perceived as offering significant benefits to AYPLHIV, including reduced pill burden, improved adherence, minimized risk for inadvertent disclosure, and reduced stigma around HIV and oral ART. Concerns have been raised about the lack of knowledge and awareness of LA ART and the potential strain it may place on health systems as healthcare providers prepare for increased workloads from managing a higher volume of patients receiving monthly or 2-monthly injections. Considerations for cost-effectiveness, waste management, and sustainability of supply chain matter to policymakers. A multi-pronged approach is needed to implement LA ART in LMICs effectively. This includes increased education and awareness campaigns for both healthcare providers and AYPLHIV, along with policy changes to bolster healthcare systems and address the strain of increased workloads associated with this promising treatment.

## Data Availability

The original contributions presented in the study are included in the article/Supplementary material, further inquiries can be directed to the corresponding author.

## References

[1] Kenya Universal Health Coverage Policy 2020-2030. P4H Network. Available on: https://p4h.world/en/documents/kenya-universal-health-coverage-policy-2020-2030/ (accessed August 29, 2024).

[2] Unintended pregnancies and HIV among adolescents and young people. UNICEF Kenya. (2020). Available on: https://www.unicef.org/kenya/reports/unintended-pregnancies-and-hiv-among-adolescents-and-young-people-Homa-Bay (accessed October 3, 2024).

[3] Mkumba LS Nassali M Benner J Ritchwood TD. Sexual and reproductive health needs of young people living with HIV in low- and middle-income countries: a scoping review. Reprod Health. (2021) 18:219. 10.1186/s12978-021-01269-7 34740379 PMC8570025

[4] UNAIDS. We've Got The Power: Women, Adolescent girls and the HIV Response [Website]. Geneva: Joint United Nations Programme on HIV/AIDS (UNAIDS) (2020). Available online at: https://www.unaids.org/sites/default/files/media_asset/2020_women-adolescent-girls-and-hiv_en.pdf

[5] UNAIDS. Co-creating a New Global Initiative to End AIDS Among Children, Adolescents, and Their Mothers [Internet]. Geneva: Joint United Nations Programme on HIV/AIDS (UNAIDS) (2021). Available online at: https://www.unaids.org/en/resources/presscentre/featurestories/2021/december/20211210_icasa-pmtct (cited Feb 21, 2025).

[6] Kenya Ministry of Health (MoH). Kenya AIDS Strategic Framework II Sustain Gains, Bridge Gaps and Accelerate Progress. Available on: https://nsdcc.go.ke/wp-content/uploads/2021/05/KASFII_Web-10-Final.pdf (accessed January 31, 2023).

[7] Hudelson C Cluver L. Factors associated with adherence to antiretroviral therapy among adolescents living with HIV/AIDS in low- and middle-income countries: a systematic review. AIDS Care. (2015) 27:805–16. 10.1080/09540121.2015.1011073 25702789

[8] Daniel AK Casmir E Oluoch L Micheni M Kiptinness C Wald A. “I was just concerned about getting pregnant”: Attitudes toward pregnancy and contraceptive use among adolescent girls and young women in Thika, Kenya. BMC Pregn Childbirth. (2023) 23:493. 10.1186/s12884-023-05802-3 37403049 PMC10321002

[9] ViiV Healthcare. ViiV Healthcare Announces US FDA Approval of Cabenuva (Cabotegravir, Rilpivirine) for Virologically Suppressed Adolescents Living With HIV Who Are 12 Years of Age or Older and Weigh at Least 35 kg [Internet]. Research Triangle Park, NC: ViiV Healthcare (2022). Available online at: https://viivhealthcare.com/en-us/media-center/news/press-releases/2022/march/viiv-healthcare-announces-us-fda-approval-of-cabenuva/ (cited Feb 21, 2025).

[10] ViiV ViiV Healthcare announces US FDA approval of Cabenuva (cabotegravir rilpivirine) rilpivirine) for virologically suppressed adolescents living with HIV who are 12 years of age or older and weigh at least 35 kg. Available on: https://viivhealthcare.com/en-us/media-center/news/press-releases/2022/march/viiv-healthcare-announces-us-fda-approval-of-cabenuva/ (accessed February 5, 2024).

[11] Benning L Mantsios A Kerrigan D Coleman JS Golub E Blackstock O. Examining adherence barriers among women with HIV to tailor outreach for long-acting injectable antiretroviral therapy. BMC Women's Health. (2020) 20:152. 10.1186/s12905-020-01011-8 32711509 PMC7382076

[12] Kerrigan D Sanchez Karver T Muraleetharan O Savage V Mbwambo J Donastorg Y. “A dream come true”: perspectives on long-acting injectable antiretroviral therapy among female sex workers living with HIV from the Dominican Republic and Tanzania. PLoS ONE. (2020) 15:e0234666. 10.1371/journal.pone.0234666 32530939 PMC7292359

[13] Kityo C Cortes CP Phanuphak N Grinsztejn B Venter F. Barriers to uptake of long-acting antiretroviral products for treatment and prevention of HIV in low- and middle-income countries (LMICs). Clin Infect Dis. (2022) 75:S549–S556. 10.1093/cid/ciac752 36410377

[14] The LATITUDE Study: Long-Acting Therapy to Improve Treatment SUccess in Daily LifE. Available on: https://www.uclahealth.org/clinical-trials/latitude-study-long-acting-therapy-improve-treatment-success (accessed October 7, 2024).

[15] Gandhi M. High virologic suppression rates on long-acting ART in a safety-net clinical population. In: 2023 Conference on Retroviruses and Opportunistic Infections (2023).37399555

[16] ClinicalTrials.gov *More Options for Children and Adolescents (MOCHA): Oral and Long-Acting Injectable Cabotegravir and Rilpivirine in HIV-Infected Children and Adolescents (MOCHA) [Internet]*. Bethesda, MD: National Library of Medicine (US) (2022). Available online at: https://clinicaltrials.gov/study/NCT03497676 (cited Feb 21, 2025).

[17] ClinicalTrials.gov *Long-Acting Treatment in Adolescents (LATA) [Internet]*. Bethesda, MD: National Library of Medicine (US) (2022). Available online at: https://clinicaltrials.gov/study/NCT05154747 (cited Feb 21, 2025).

[18] Kerrigan D Mantsios A Gorgolas M Montes M-L Pulido F Brinson C. Experiences with long acting injectable ART: a qualitative study among PLHIV participating in a Phase II study of cabotegravir + rilpivirine (LATTE-2) in the United States and Spain. PLoS ONE. (2018) 13:e0190487. 10.1371/journal.pone.0190487 29304154 PMC5755771

[19] Jolayemi O Bogart LM Storholm ED Goodman-Meza D Rosenberg-Carlson E Cohen R. Perspectives on preparing for long-acting injectable treatment for HIV among consumer, clinical and nonclinical stakeholders: a qualitative study exploring the anticipated challenges and opportunities for implementation in Los Angeles County. PLoS ONE. (2022) 17:e0262926. 10.1371/journal.pone.0262926 35113892 PMC8812879

[20] Simoni JM Beima-Sofie K Mohamed ZH Christodoulou J Tapia K Graham SM. Long-acting injectable antiretroviral treatment acceptability and preferences: a qualitative study among US providers, adults living with HIV, and parents of youth living with HIV. AIDS Patient Care STDS. (2019) 33:104–11. 10.1089/apc.2018.0198 30844308 PMC6442271

[21] Philbin MM Parish CL Kinnard EN Reed SE Kerrigan D Alcaide ML. Multisite study of women living with HIV's perceived barriers to, and interest in, long-acting injectable antiretroviral therapy. J Acquir Immune Defic Syndr. (2020) 84:263–70. 10.1097/QAI.0000000000002337 32530905 PMC7483266

[22] Mantsios A Murray M Karver TS Davis W Galai N Kumar P. Multi-level considerations for optimal implementation of long-acting injectable antiretroviral therapy to treat people living with HIV: perspectives of health care providers participating in phase 3 trials. BMC Health Serv Res. (2021) 21:255. 10.1186/s12913-021-06214-9 33743684 PMC7980753

[23] Toska E Zhou S Chen-Charles J Gittings L Operario D Cluver L. Factors associated with preferences for long-acting injectable antiretroviral therapy among adolescents and young people living with HIV in South Africa. AIDS Behav. (2023) 27:2163–2175. 10.1007/s10461-022-03949-2 36622486 PMC9827015

[24] Zakumumpa H Alinaitwe A Kyomuhendo M Nakazibwe B. Long-acting injectable antiretroviral treatment: experiences of people with HIV and their healthcare providers in Uganda. BMC Infect Dis. (2024) 24:876. 10.1186/s12879-024-09748-5 39198739 PMC11360315

[25] Kenya Ministry of Health. County HIV Estimates Workbook. (2023). Available on: https://analytics.nsdcc.go.ke/estimates/#!/kenya/countywkbook (accessed July 22, 2024).

[26] Four teens infected with HIV weekly in Uasin Gishu — report. The Star. Available on: https://www.the-star.co.ke/news/2022-11-18-four-teens-infected-with-hiv-weekly-in-uasin-gishu-report/ (accessed February 5, 2024).

[27] Tong A Sainsbury P Craig J. Consolidated criteria for reporting qualitative research (COREQ): a 32-item checklist for interviews and focus groups. Int J Qual Health Care. (2007) 19:349–57. 10.1093/intqhc/mzm042 17872937

[28] The Social-Ecological Model: A Framework for Prevention |Violence Prevention|Injury Center|CDC. (2022). Available on: https://www.cdc.gov/violenceprevention/about/social-ecologicalmodel.html (accessed February 5, 2024).

[29] Kilanowski JF. Breadth of the socio-ecological model. J Agromedicine. (2017) 22:295–297. 10.1080/1059924X.2017.1358971 28742433

[30] Feldstein AC Glasgow RE. A practical, robust implementation and sustainability model (PRISM) for integrating research findings into practice. Jt Comm J Qual Patient Saf. (2008) 34:228–43. 10.1016/S1553-7250(08)34030-6 18468362

[31] Braun CV V. Using thematic analysis in psychology. Qualit Res Psychol. (2006) 3:77–101. 10.1191/1478088706qp063oa32100154

[32] Braun V Clarke V. What can “thematic analysis” offer health and wellbeing researchers? Int J Qual Stud Health Well-being. (2014) 9:26152. 10.3402/qhw.v9.26152 25326092 PMC4201665

[33] Chounta V Overton ET Mills A Swindells S Benn PD Vanveggel S. Patient-reported outcomes through 1 year of an HIV-1 clinical trial evaluating long-acting cabotegravir and rilpivirine administered every 4 or 8 weeks (ATLAS-2M). Patient. (2021) 14:849–62. 10.1007/s40271-021-00524-0 34056699 PMC8563641

[34] Cooper SE Rosenblatt J Gulick RM. Barriers to uptake of long-acting antiretroviral products for treatment and prevention of human immunodeficiency virus (HIV) in high-income countries. Clin Infect Dis. (2022) 75:S541–8. 10.1093/cid/ciac716 36410385 PMC10200323

[35] Rosen S Grimsrud A Ehrenkranz P Katz I. Models of service delivery for optimizing a patient's first six months on antiretroviral therapy for HIV: an applied research agenda. Gates Open Res. (2020) 4:116. 10.12688/gatesopenres.13159.1 32875281 PMC7445417

[36] Rosen S Nichols B Guthrie T Benade M Kuchukhidze S Long L. Do differentiated service delivery models for HIV treatment in sub-Saharan Africa save money? Synthesis of evidence from field studies conducted in sub-Saharan Africa in 2017-2019. Gates Open Res. (2022) 5:177. 10.12688/gatesopenres.13458.2 35310814 PMC8907143

[37] De Neve J-W Boudreaux C Gill R Geldsetzer P Vaikath M Bärnighausen T. Harmonizing community-based health worker programs for HIV: a narrative review and analytic framework. Hum Resour Health. (2017) 15:45. 10.1186/s12960-017-0219-y 28673361 PMC5496353

[38] Pallas SW Minhas D Pérez-Escamilla R Taylor L Curry L Bradley EH. Community health workers in low- and middle-income countries: what do we know about scaling up and sustainability? Am J Public Health. (2013) 103:e74–82. 10.2105/AJPH.2012.301102 23678926 PMC3682607

[39] de Vries DH Pool R. The influence of community health resources on effectiveness and sustainability of community and lay health worker programs in lower-income countries: a systematic review. PLoS ONE. (2017) 12:e0170217. 10.1371/journal.pone.0170217 28095475 PMC5240984

[40] *Injectable HIV therapy would have to cost less than $131 a year to be cost-effective in Africa*. aidsmap.com. (2021). Available on: https://www.aidsmap.com/news/apr-2021/injectable-hiv-therapy-would-have-cost-less-131-year-be-cost-effective-africa (accessed October 7, 2024).

[41] Cresswell FV Lamorde M. Implementation of long-acting antiretroviral therapy in low-income and middle-income countries. Curr Opin HIV AIDS. (2022) 17:127–34. 10.1097/COH.0000000000000732 35439787

[42] Chisholm JM Zamani R Negm AM Said N Daiem MMA Dibaj M. Sustainable waste management of medical waste in African developing countries: a narrative review. Waste Manag Res. (2021) 39:1149–63. 10.1177/0734242X211029175 34218734 PMC8488638

[43] Czarnogorski M. Customize: overall results from a hybrid III implementation-effectiveness study examining implementation of cabotegravir and rilpivirine long-acting injectable for HIV treatment in US healthcare settings; final patient and provider data. In: 11th International AIDS Society Conference on HIV Science. (2021).

[44] Deeks SG Lewin SR Havlir DV. The end of AIDS: HIV infection as a chronic disease. Lancet. (2013) 382:1525–33. 10.1016/S0140-6736(13)61809-7 24152939 PMC4058441

[45] Echenique M Rodriguez VJ LaCabe RP Privette CK Jones DL Potter JE. Behaviorally and perinatally HIV-infected young women: targets for preconception counseling. AIDS Care. (2017) 29:372–7. 10.1080/09540121.2016.1220483 27535165 PMC5262535

